# Effects of computerized cognitive training on functional brain networks in patients with vascular cognitive impairment and no dementia

**DOI:** 10.1111/cns.14779

**Published:** 2024-06-03

**Authors:** Qiong‐Ge Li, Yi Xing, Zu‐De Zhu, Xiao‐Lu Fei, Yi Tang, Jie Lu

**Affiliations:** ^1^ Department of Radiology and Nuclear Medicine, Xuanwu Hospital Capital Medical University Beijing China; ^2^ Beijing Key Laboratory of Magnetic Resonance Imaging and Brain Informatics Beijing China; ^3^ Department of Neurology, Xuanwu Hospital Capital Medical University Beijing China; ^4^ Collaborative Innovation Center for Language Ability Jiangsu Normal University Xuzhou China; ^5^ Department of Information, Xuanwu Hospital Capital Medical University Beijing China

**Keywords:** computerized cognitive training, functional connectivity, functional magnetic resonance imaging, intrinsic connectivity network, vascular cognitive impairment no dementia

## Abstract

**Aims:**

Previous neuroimaging studies of vascular cognitive impairment, no dementia (VCIND), have reported functional alterations, but far less is known about the effects of cognitive training on functional connectivity (FC) of intrinsic connectivity networks (ICNs) and how they relate to intervention‐related cognitive improvement. This study provides comprehensive research on the changes in intra‐ and inter‐brain functional networks in patients with VCIND who received computerized cognitive training, with a focus on the underlying mechanisms and potential therapeutic strategies.

**Methods:**

We prospectively collected 60 patients with VCIND who were randomly divided into the training group (*N* = 30) receiving computerized cognitive training and the control group (*N* = 30) receiving fixed cognitive training. Functional MRI scans and cognitive assessments were performed at baseline, at the 7‐week training, and at the 6‐month follow‐up. Utilizing templates for ICNs, the study employed a linear mixed model to compare intra‐ and inter‐network FC changes between the two groups. Pearson correlation was applied to calculate the relationship between FC and cognitive function.

**Results:**

We found significantly decreased intra‐network FC within the default mode network (DMN) following computerized cognitive training at Month 6 (*p* = 0.034), suggesting a potential loss of functional specialization. Computerized training led to increased functional coupling between the DMN and sensorimotor network (SMN) (*p* = 0.01) and between the language network (LN) and executive control network (ECN) at Month 6 (*p* < 0.001), indicating compensatory network adaptations in patients with VCIND. Notably, the intra‐LN exhibited enhanced functional specialization after computerized cognitive training (*p* = 0.049), with significant FC increases among LN regions, which correlated with improvements in neuropsychological measures (*p* < 0.05), emphasizing the targeted impact of computerized cognitive training on language abilities.

**Conclusions:**

This study provides insights into neuroplasticity and adaptive changes resulting from cognitive training in patients with VCIND, with implications for potential therapeutic strategies.

## Graphical abstract

Computerized cognitive training enhances cognition in patients with VCIND and induces alterations in the overall brain network structure. These modifications suggest a comprehensive reconstruction in information flow across brain networks, with notable increases in functional connectivity within the LN regions. The strengthened LN connections correlate with improved neuropsychological measures, underscoring the focused influence of cognitive training on language abilities.

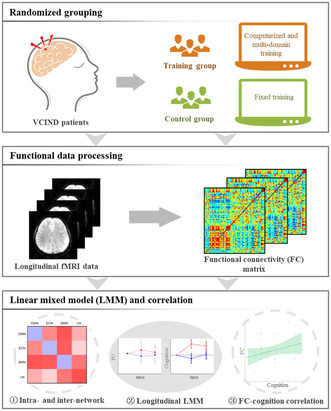

## 1 INTRODUCTION

Vascular cognitive impairment, no dementia (VCIND) refers to a condition characterized by cognitive impairments that are caused by vascular damage in the brain but do not meet the criteria for dementia.^1^, ^2^ VCIND is often considered a milder form of vascular cognitive impairment (VCI) than vascular dementia.^3^, ^4^ According to epidemiological research, the prevalence of VCI among the population aged 65 and above in China is approximately 8.7%,^5^ which is an important public health issue. VCIND, as an early stage of VCI, is closely associated with declining cognitive abilities and an increased risk of developing dementia.^6^, ^7^ Therefore, it is important to note that while VCIND does not meet the criteria for dementia, individuals with this condition are at an increased risk of developing dementia in the future.

Proper management and treatment are crucial for VCIND to prevent further cognitive decline. Most studies have shown that targeted cognitive training for elderly individuals can maintain or even enhance cognitive function in specific domains.^8^, ^9^ Nevertheless, certain researchers have proposed that the intervention impact of singular, standardized cognitive training for neurocognitive disorders, including early Alzheimer's disease (AD), is restricted. High‐quality cognitive training and cognitive rehabilitation assessments, on the other hand, have shown substantial efficacy.^10^, ^11^ Computerized cognitive training for early‐stage dementia patients and individuals with mild cognitive impairment (MCI) has also been proven to be an effective intervention.^12^, ^13^ In recent years, research has proposed systematic cognitive training for VCIND patients, which has shown significant improvements in visuospatial function, memory function, language function, and attention function while partially improving execution function.^14^ In addition, a multicenter VCIND study, employing a randomized, active controlled design using multidomain, adaptive, computerized cognitive training, further confirmed that computerized cognitive training can significantly improve language function, execution function, and overall cognitive function in patients.^15^, ^16^ However, the underlying brain mechanisms associated with changes in cognitive function still require further investigation.

Neuroimaging has been proven to provide information about changes in brain structure and function, as well as the relationship between these changes and accompanying cognitive changes.^17^ Resting‐state functional magnetic resonance imaging (FMRI) is a technique that allows for rapid brain functional imaging without the need for task stimulation. It can construct multiple reproducible intrinsic connectivity networks (ICNs),^18^, ^19^ where the default mode network (DMN), executive control network (ECN), and sensorimotor network (SMN) are among the key functional networks associated with cognitive function.^20^, ^21^ ICNs refer to the complex connections and interactions between different brain regions that are responsible for cognitive processes, such as memory, language, motor, and executive function, which are highly suitable for studying changes in brain function in patients with VCIND after cognitive training.

ICNs consist of both intra‐network connections that represent specific functional brain networks and inter‐network connections that reflect functional coupling between different brain networks. These two components complement each other. The changes in brain functional networks in patients with VCIND may provide insights into the mechanisms of cognitive impairment and could help to develop effective therapeutic strategies for this condition. Recent studies have investigated the changes in brain functional brain networks in patients with VCIND to gain a better understanding of the underlying mechanisms of the disease. Several studies have reported alterations in functional connectivity (FC), particularly in the DMN, which is a network of brain regions that is active when the brain is at rest and not engaged in any task.^22^, ^23^ The DMN is involved in a range of cognitive functions, including memory, self‐referential processing, and social cognition, and its dysfunction has been implicated in VCIND and several neurodegenerative disorders.^19^ In addition, research has found that the executive function and language function of VCIND patients undergo change as the disease progresses.^24^, ^25^ Cerebrovascular damage often leads to abnormalities in sensory‐motor functions.^26^ The ICNs corresponding to executive function, language function, and sensory‐motor function are referred to as the ECN, language network (LN), and SMN, respectively. Furthermore, studies have proposed significant improvements in executive function and language function in patients with VCIND after cognitive training.^14^, ^15^ These findings suggest that the changes in the DMN, LN, ECN, and SMN in VCIND play an important role in studying the integration and disruption of brain function in patients with VCIND undergoing cognitive training.

Although there have been studies investigating the brain functional network mechanisms in patients with VCIND, longitudinal tracking studies specifically focusing on cognitive training interventions for the disease are still lacking. Thus, this article aims to provide comprehensive research on the changes in intra‐ and inter‐brain functional networks in patients with VCIND who received computerized cognitive training, with a focus on the underlying mechanisms and potential therapeutic strategies. Here, we examined and compared longitudinal intra‐ and inter‐network FC changes in a group of patients with VCIND who received computerized cognitive training and another group who received a fixed training program. We focused on FC within and between four ICNs (DMN, ECN, SMN, and LN) and their relationships with cognitive performance. We hypothesized that patients with VCIND with computerized cognitive training would be characterized by improved topological features of functional networks (both intra‐network and inter‐network).

## 2 METHODS

### 2.1 Participants

**FIGURE 1:**
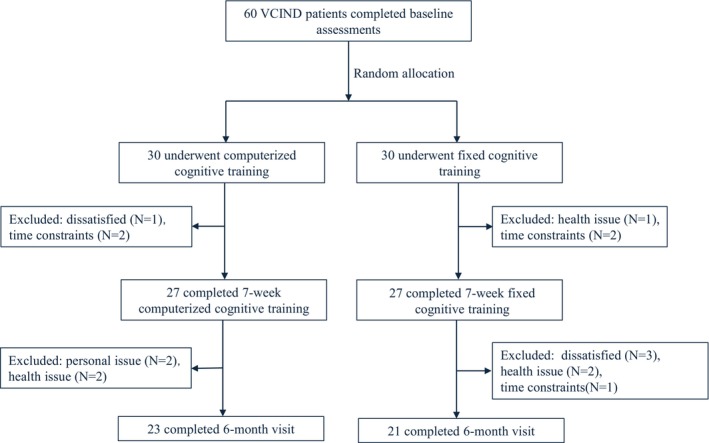
Flowchart of the enrolled and followed subjects in the control group and training group during the three timepoints of the study. The control group and the training group included 30 participants who received 7 weeks of cognitive training and a 6‐month visit. Several patients from the dataset were discarded from the cohorts because they had no MRI or neuropsychological recordings at that time.

We recruited 60 patients with VCIND, who were diagnosed with cognitive impairment without dementia and small vessel ischemia by a consensus panel including three senior neurologists. These 60 participants were then randomly assigned to either a training group or a control group, the personnel involved in conducting the study and data analysis were masked to the patient randomization. Study participants, their caregivers, and all assessors were blinded to treatment assignment throughout the study. Both groups were followed up three times over 6 months (Figure 1).

Patients in the training group received a series of computerized training domains, including processing speed, attention, perception, long‐term memory, working memory, calculation, executive control, reasoning, and problem‐solving. Tasks included various exercises like number and object detection, letter and chess selection, dual‐attending tasks, and memory exercises such as the one‐back and n‐back tasks. Participants completed 30‐minute daily sessions with tasks tailored to individual performance levels. In each training domain, the training accuracy of the patients is greater than 80% to advance to the next level of difficulty. The control group underwent training with tasks focused solely on processing speed and attention domains, maintained at a fixed and primary difficulty level throughout the study. These tasks were completed for the same duration as those in the training group. The whole advanced training of the two groups lasted for 7 weeks.

The design of this study followed the CONSORT statement and the CONSORT statement of non‐pharmacological intervention. The study was approved by the local Ethics Committee. The trial was registered under ClinicalTrail.gov (NCT02640716). All participants provided written informed consent before participation.

### 2.2 Neuropsychological assessment

All participants underwent three neuropsychological assessments by trained researchers at baseline, 7 weeks (i.e., the end of intervention), and 6 months after training to measure the effect of the intervention. Three cognitive domains were evaluated^27^: global cognitive function measured by the Montreal Cognitive Assessment (MoCA), executive function measured by the Trail Making Test B‐A (TMT B‐A), and linguistic function by the Boston Naming Test (BNT). The scores of each test at each time point were standardized to T scores concerning the baseline.

### 2.3 Image acquisition

Magnetic resonance imaging (MRI) scans were conducted on a 3.0 T Siemens Tim Trio System (Siemens, Erlangen, Germany). All participants performed resting‐state fMRI scans using a multiband echo‐planar imaging (EPI) sequence: 35 continuous axial slices, repetition time (TR) = 2000 ms, echo time (TE) = 30 ms, flip angle =83°, field of view (FOV) = 224 × 224 m^2^, voxel size = 3.5 × 3.5 × 3.5 mm^3^. The scan lasted 328 seconds and needed 164 volumes. High‐resolution T1‐weighted structural MRI was acquired using a magnetization‐prepared rapid gradient echo (MPRAGE) sequence: 192 continuous sagittal slices, TR/TE = 1690/2.56 ms, flip angle = 12°, FOV = 256 × 256 mm^2^, voxel size = 1.0 × 1.0 × 1.0 mm^3^. All participants underwent three MRI scans at baseline, at the end of the intervention, and 6 months after training to measure the effect of the intervention.

### 2.4 Image preprocessing

All rs‐fMRI data were preprocessed with the Statistical Parametric Mapping (SPM12; Wellcome Department of Imaging Neuroscience, London, UK) package and Data Processing Assistant for Resting‐State fMRI (DPARSF) toolkit.^28^ First of all, quality control measures were implemented to ensure data integrity and reliability, including visual inspection of images, assessment of motion artifacts, and evaluation of signal‐to‐noise ratio. After removing the first four volumes, we subjected the remaining volumes to (1) slice‐time correction, (2) realign for head motion correction, (3) spatial normalization to the Montreal Neurological Institute (MNI) space with a voxel size of 3 × 3 × 3 mm^3^, (4) smoothing with a 4‐mm full‐width half‐maximum (FWHM) Gaussian kernel, (5) removal of several nuisance variables (including Friston's 24 head‐motion parameters and averaged signals from white matter and cerebrospinal fluid tissue) using multivariate linear regression analysis^29^, ^30^ and (6) temporal filtering with 0.01–0.1 Hz bandpass.

### 2.5 Brain network construction

**FIGURE 2:**
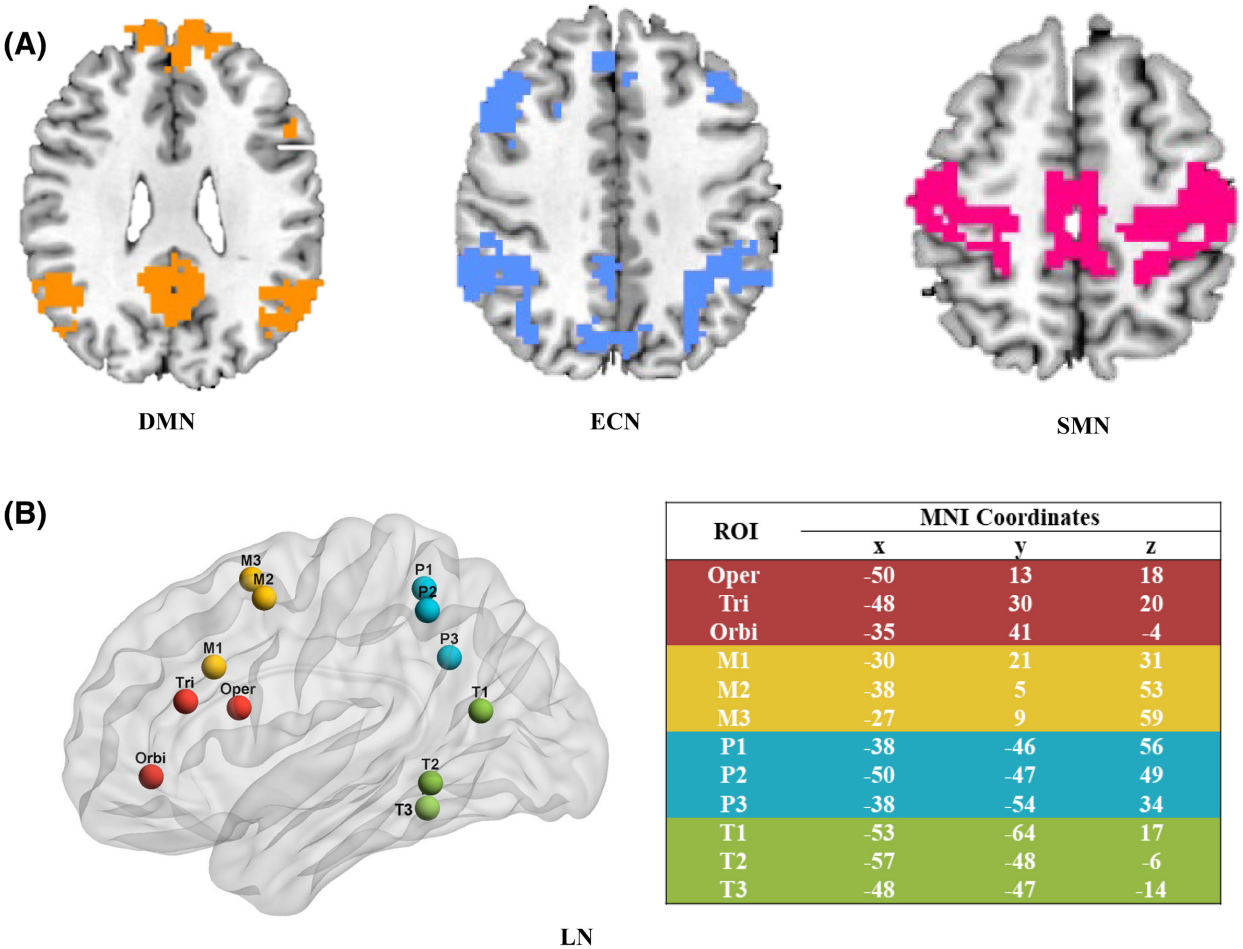
(A) Locations of DMN, ECN, and SMN. (B) Detailed ROIs of LN utilized in the G network analysis. Red represents the ROIs located in the Broca region, green represents the ROIs located in the temporal lobe, yellow represents the ROIs located in the medial frontal gyrus, and blue represents the parietal lobe. DMN, default mode network; ECN, executive control network; LN, language network; M1, the anterior inferior part of the middle frontal gyrus; M2, the posterior superior part of the middle frontal gyrus; M3, the middle frontal gyrus area between M1 and M2; MNI, Montreal Neurological Institute; Oper, pars opercularis; Orbi, pars orbitalis; P1, superior and anterior part of the superior and inferior parietal lobules; P2, the area adjacent to and overlapping with the angular gyrus; P3 is the area between P1 and P2 in the superior and inferior parietal lobules; SMN, sensorimotor network; T1, the posterior superior temporal gyrus, and the superior middle temporal gyrus; T2, the inferior middle temporal gyrus; T3, inferior superior temporal gyrus; Tri, pars triangularis.

We focused on four functional brain networks: the DMN, ECN, SMN, and LN (Figure 2). The first three networks were spatially defined by the parcellation templates based on the study of Yeo et al.,^31^ and the LN‐defined regions of interest (ROIs) were adopted from previous studies conducted by Skeide et al.^32^ The detailed MNI coordinates of LN are shown in Figure 2B. Finally, a total of 72 ROIs were isolated. At the subject level, FC (FC) between each pair of cortical ROIs was computed as Pearson's correlation coefficient (*r* value). Each *r* value was then Fisher's r‐to‐z transformed. A continuous network G (72 × 72) for each subject was then constructed for each study time point. Average z scores of intra‐networks (DMN, ECN, SMN, and LN) and inter‐network FC (ECN–DMN, ECN–SMN, ECN‐LN, DMN–SMN, DMN‐LN, SMN‐LN) were then calculated for each participant at each visit for further statistical analyses.

The quality control process was carried out for selecting regions of interest (ROIs) within the LN to ensure reliability and validity. First, anatomical landmarks and FC patterns from established literature were used to guide ROI selection. Second, each ROI was visually inspected to confirm accurate delineation and placement within the targeted brain regions. Third, FC maps were examined to verify that selected ROIs exhibited robust and consistent connectivity patterns with other language‐related regions. Finally, inter‐rater reliability checks were performed to assess the consistency of ROI identification across different raters or imaging analysts. These quality control measures were implemented to enhance the validity and reproducibility of ROI selection within the LN.

### 2.6 Statistical analysis

Before analysis, the normality of data distribution was assessed using Shapiro–Wilk tests. For each variable, we performed nonparametric equivalent tests if the data did not exhibit a normal distribution. For characteristics and training adherence of the two groups in the baseline period, independent sample *t*‐tests and chi‐square tests were used for statistical analysis of measurement data and count data, respectively. To examine the effects of cognitive training, we modeled the longitudinal changes in neuropsychological scores and connectivity parameters using a linear mixed model (LMM).^33^ Time was assigned as the repeated variable. Group, time, and group‐by‐time were included as fixed effects (two tailed, *p* < 0.05). Correlation analyses between significant brain functional changes and neuropsychological scores were then performed to explore a potential neural mechanism for cognitive functional changes.

### 2.7 Validation analysis

We performed a validation analysis to test the robustness of our results. We verified the results by In addition employing local efficiency and global efficiency. For validation analysis, we used the sparsity threshold S to define the regime (0.10 < S < 0.50). Each topological attribute was compared with those of 100 random networks to produce a normalized value, and areas under the curve (AUC) were calculated for statistical comparisons. Refer to the Data S1 for detailed algorithms.

## 3 RESULTS

### 3.1 Demographics

Demographic characteristics and main neuropsychological information are shown in Table 1. No significant differences in age, sex, or education were found between the two groups (*p* > 0.05). At baseline, the two groups of patients with VCIND also showed no significant differences in the MoCA, TMT B‐A, or BNT scores (*p* < 0.05).

**TABLE 1:** Baseline demographic and neuropsychological assessment for VCIND patients.

Variables	Training group *N* = 30	Control group *N* = 30	t/χ^2^	*p*
Age, years	65.93 ± 7.89	64.93 ± 6.66	0.53	0.60
Male/Female	18/12	22/8	1.20	0.42
Education, years	10.87 ± 3.53	10.03 ± 2.85	1.01	0.32
MoCA	21.90 ± 3.83	21.23 ± 3.85	0.66	0.51
TMT B‐A	75.18 ± 57.25	77.00 ± 65.35	−0.11	0.91
BNT	22.17 ± 3.82	23.46 ± 3.59	−1.32	0.19

*Note*: Independent sample *t* tests were used for age, education and neuropsychological tests; chi‐squared tests were used for sex comparisons.

Abbreviations: BNT, Boston Naming Test; MoCA, Montreal Cognitive Assessment; TMT B‐A, Trail Making Test B‐A; VCIND, vascular cognitive impairment, no dementia.

### 3.2 Longitudinal neuropsychological changes

As shown in Table 2, MoCA and BNT in the training group exhibited significant improvements at Week 7 (*p* < 0.05), and there were no significant changes at Month 6. Compared with the control group, TMT B‐A showed no significant group × time interaction during the two visits.

**TABLE 2:** Longitudinal neuropsychological changes in the training group and control group.

Variables	Change from baseline to Week 7			Change from the baseline to Month 6		
	Training group (95% CI)	Control group (95% CI)	*p* (group × time)	Training group (95% CI)	Control group (95% CI)	*p* (group × time)
MoCA	3.356 (1.467 to 5.244)	−0.085 (−2.062 to 1.892)	**0.013**	2.224 (0.256–4.192)	1.358 (20.899 to 3.614)	0.562
TMT B‐A	−5.958 (−38.558 to 26.642)	−10.702 (−45.259 to 23.856)	0.842	−3.891 (−37.031 to 29.249)	−17.596 (−51.693 to 16.500)	0.564
BNT	2.500 (0.595 to 4.405)	20.687 (22.845 to 1.472)	**0.028**	2.015 (20.026 to 4.056)	−0.192 (−2.308 to 1.925)	0.135

*Note*: The bold values indicate statistically significant differences between groups (*p* < 0.05).

Abbreviations: BNT, Boston Naming Test; MoCA, Montreal Cognitive Assessment; TMT B‐A, Trail Making Test B‐A.

### 3.3 Longitudinal changes in intra‐network and inter‐network FC

**FIGURE 3:**
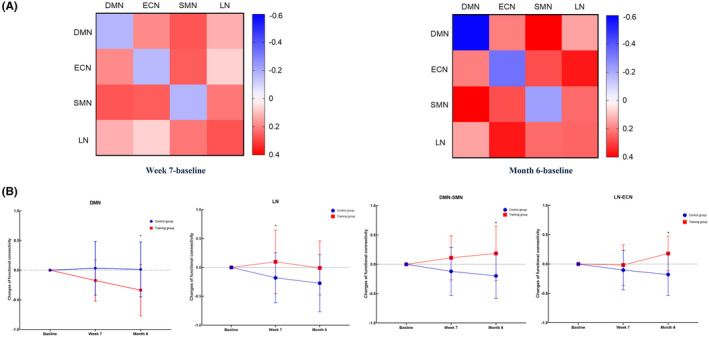
(A) Heatmap showing differences of intra‐network and inter‐network FC between the training group and control group at week‐baseline (left) and Month 6‐baseline (right). Warmer colors represent increased FC, while cooler colors represent decreased FC. (B) Cognitive training‐dependent changes in intra‐network functional connectivity in the DMN, LN, and inter‐network functional connectivity between the DMN and SMN and between the LN and ECN. *Marks the significant group × time effect.

We observed significant longitudinal decreases in intra‐network FC within the DMN at Month 6 (*p* = 0.034) and longitudinal increases in LN at Week 7 (*p* = 0.049) in the training group (Figure 3 and Table 3). Although there were decreasing FC trends in the intra‐ECN and intra‐SMN in the training group, these results did not reach statistical significance (Figure 3). These findings indicate reduced functional specialization of the DMN, ECN, and SMN and improved functional specialization of the LN in VCIND patients with advanced training.

**TABLE 3:** Longitudinal functional connectivity changes of intra‐ and inter‐networks for the training group and control group.

Variables	Change from baseline to Week 7			Change from the baseline to Month 6		
	Training group (95% CI)	Control group (95% CI)	*p* (group × time)	Training group (95% CI)	Control group (95% CI)	*p* (group × time)
Intra‐DMN	−0.151 (−0.408 to 0.073)	0.022 (−0.180 to 0.543)	0.122	−0.411 (−0.674 to 0.051)	0.577 (−0.314 to 0.372)	**0.034**
Intra‐LN	0.118 (−0.305 to 0.345)	−0.151 (−0.394 to 0.126)	**0.049**	0.098 (−0.504 to 0.345)	−0.180 (−0.469 to −0.093)	0.070
DMN‐SMN	0.155 (−0.228 to 0.410)	−0.100 (−0.380 to 0.160)	0.086	0.190 (−0.105 to 0.529)	−0.210 (−0.395 to 0.410)	**0.010**
LN‐ECN	0.012 (−0.306 to 0.291)	−0.060 (−0.366 to 0.176)	0.373	0.155 (−0.060 to 0.437)	−0.210 (−0.366 to 0.176)	**0.000**

*Note:* The bold values indicate statistically significant differences between groups (*p* < 0.05). Abbreviations: DMN, default mode network; ECN, executive control network; LN, language network; SMN, sensorimotor network.

Modeling inter‐network FC between each of the four ICNs (DMN–ECN, DMN–SMN, DMN‐LN, LN‐ECN, LN–SMN, ECN–SMN), we observed an overall FC increasing trend in inter‐network in the training group. Specifically, there were significantly increased training‐dependent FC changes in the DMN–SMN (*p* = 0.01) and LN‐ECN (*p* < 0.001) at Month 6 in the training group, showing stronger functional coupling with computerized cognitive training in patients with VCIND. (Figure 3 and Table 3).

### 3.4 Longitudinal changes in LN

**FIGURE 4:**
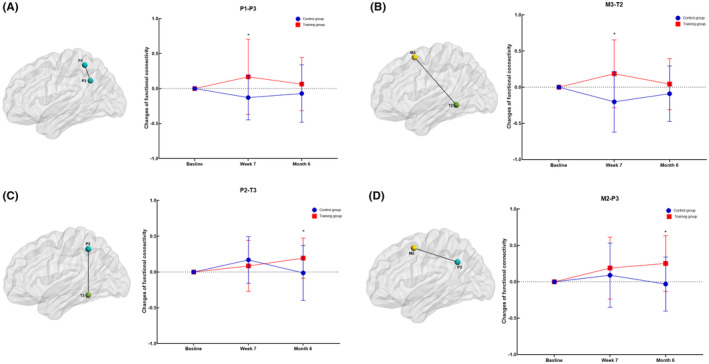
Cognitive training‐dependent FC changes in the LN. (A), P1‐P3. (B), M3‐T2. (C), P2‐T3, (D), M2‐P3. *Marks the significant group × time effect.

We found robust longitudinal FC increases in P1‐P3 (*p* = 0.019) and M3‐T2 (*p* = 0.002) in the training group at Week 7. In addition, there were also significant FC increases in P2‐T3 (*p* = 0.021) and M2‐P3 (*p* = 0.001) at Month 6 (Figure 4 and Table 4).

**TABLE 4:** Longitudinal functional connectivity changes in the LN for the training group and control group.

Variables	Change from baseline to Week 7			Change from the baseline to Month 6		
	Training group (95% CI)	Control group (95% CI)	*p* (group × time)	Training group (95% CI)	Control group (95% CI)	*p* (group × time)
P1–P3	0.171 (−0.149 to 0.257)	−0.093 (−0.361 to 0.107)	**0.019**	0.036 (−0.220 to 0.421)	−0.094 (−0.386 to −0.257)	0.562
M3–T2	0.244 (−0.101 to 0.451)	−0.218 (−0.484 to 0.192)	**0.002**	−0.002 (−0.205 to 0.352)	−0.044 (−0.345 to 0.130)	0.564
P2–T3	−0.033 (−0.122 to 0.327)	0.244 (−0.036 to 0.398)	0.332	0.246 (−0.044 to −0.478)	−0.081 (−0.287 to 0.166)	**0.021**
M2–P3	0.131 (−0.093 to 0.487)	0.151 (−0.169 to 0.410)	0.481	0.226 (−0.022 to 0.498)	0.011 (−0.285 to 0.226)	**0.010**

*Note:* The bold values indicate statistically significant differences between groups (*p* < 0.05). Abbreviations: M2, the posterior superior part of the middle frontal gyrus; M3, the middle frontal gyrus area between the anterior inferior part of the middle frontal gyrus and M2; P1, superior and anterior part of the superior and inferior parietal lobules; P2, the area adjacent to and overlapping with the angular gyrus; P3, the area between P1 and P2 in the superior and inferior parietal lobules; T2, the inferior middle temporal gyrus; T3, inferior superior temporal gyrus.

### 3.5 Association between changes in FC and neuropsychological changes

**FIGURE 5:**
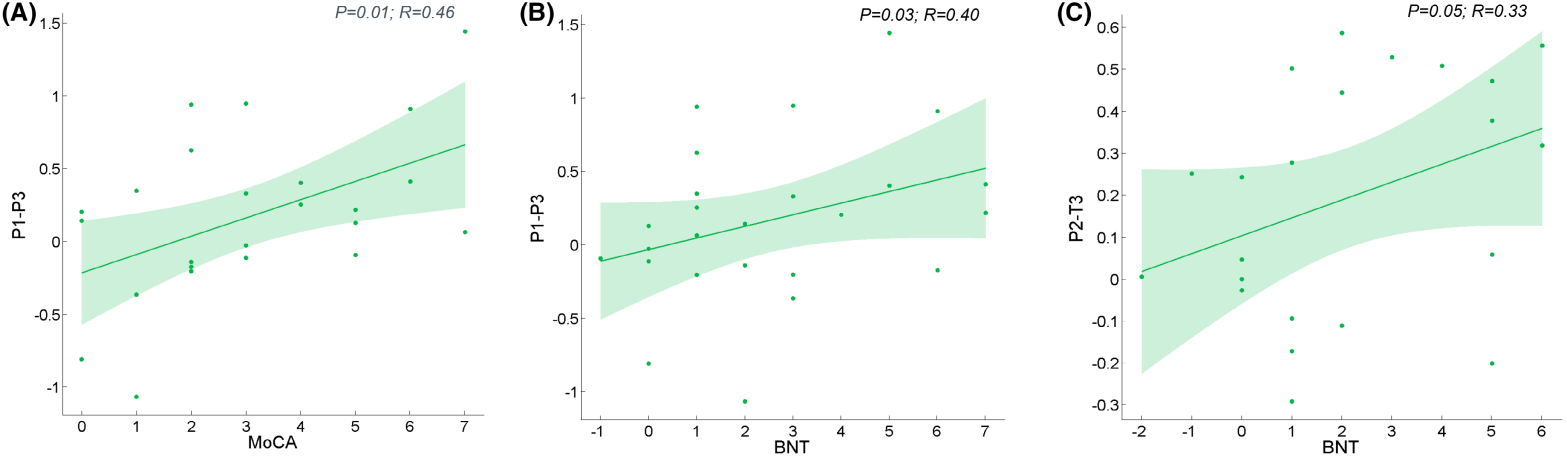
Associations between longitudinal changes in MoCA and FC measures between P1 and P3 at Week 7 (A), BNT and FC measures between P1 and P3 at Week 7 (B), and BNT with FC measures between P1 and T3 at Month 6 (C). Spaghetti plot of model‐fitted longitudinal changes in FC with training for each individual.

Based on the presence of longitudinal group × time effects, brain–neuropsychology regressions were conducted, associating the longitudinal change in neuropsychological changes with the FC changes. In the training group, there were significant associations between longitudinal changes in MoCA and FC measures between P1 and P3 at Week 7 (*p* = 0.01, *R* = 0.46), BNT and FC measures between P1 and P3 at Week 7 (*p* = 0.03, *R* = 0.40), and BNT with FC measures between P1 and T3 at Month 6 (*p* = 0.05, *R* = 0.33) (Figure 5). No other cognitive domains showed significant effects on intra‐ and inter‐networks FC.

### 3.6 Validation results

**FIGURE 6:**
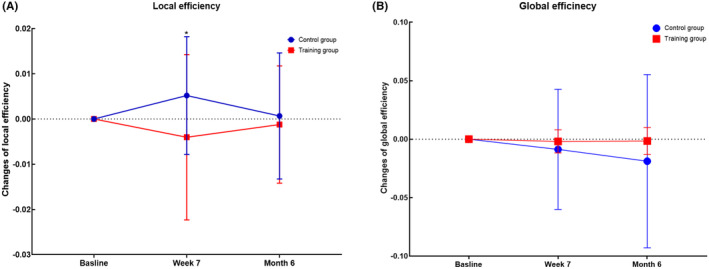
Cognitive training‐dependent changes in local efficiency (A) and global efficiency (B). *Marks the significant group × time effect.

Analysis of two confirmatory metrics (local efficiency and global efficiency) confirmed our primary findings regarding functional specialization and coupling. Specifically, as shown in Figure 6 and Table S1, the training group showed significantly lower local efficiency values (*p* = 0.030) at Week 7 and an increasing trend in global efficiency (*p* > 0.05).

## 4 DISCUSSION

The present study revealed improved cognition and altered global brain network architecture accompanied by local changes suggestive of wide‐scale reconstruction in information flow across brain networks of patients with VCIND after computerized cognitive training. Inconsistent with our hypothesis, the results exhibited advanced training‐related decreases in intra‐network FC within the DMN, likely signifying a loss of functional specialization. The increased trajectory of advanced training‐related functional coupling between the DMN and SMN, as well as between the LN and ECN, suggests compensatory efforts of networks in patients with VCIND after computerized cognitive training. In line with these results, we observed that the patients in the training group exhibited a significant decrease in local efficiency and a rising trend in global efficiency, which also indicates a reduction in the degree of functional specialization and an increase in the level of functional integration with computerized cognitive training. Of note, different from the other intra‐network FC results, the intra‐LN showed an increase in functional specialization of computerized cognitive training, and FC between the ROIs contained in the LN also showed significant advanced training‐related increasing trends, which were associated with neuropsychological changes, reflecting the important impact of cognitive training on LN function.

### 4.1 Separation and integration of brain networks

In the brain, the balance between the segregation of specialized systems and integration across systems is essential for efficient information processing and rapid information transfer within and between networks.^34^ Computerized cognitive training in patients with VCIND can lead to changes in brain network connectivity, including reduced specialization in certain networks and increased coordination between others. These adaptations may reflect the brain's plasticity and its ability to reorganize in response to cognitive challenges. Understanding these changes can help guide future interventions for cognitive disorders such as VCIND.

The study observed significant longitudinal decreases in intra‐network FC within the DMN at the 6‐month visit in patients with VCIND with computerized cognitive training. In addition, patients with computerized cognitive training exhibited longitudinal decreasing trends in intra‐ECN and intra‐SMN FC. These findings suggest that computerized cognitive training in patients with VCIND led to reduced functional specialization of the DMN, ECN, and SMN, which may indicate a shift in cognitive processing strategies. These trends in decreased FC within these networks might reflect a redistribution of cognitive resources due to computerized cognitive training.^35^ A study of cognitive training in older rats also found that FC in some brain regions decreased after the intervention, a result that could be attributed to the instability of resting brain network connections during cognitive training, which in turn delayed subjects' ability to reach optimal performance, or could be caused by greater network plasticity.^36^ In this study, we suggest that the observed decrease in FC in larger‐scale intra‐networks is a result of greater plasticity and reorganization of the brain network. Our results suggest that with computerized cognitive training, as we will discuss in the following paragraph, global network connectivity will take precedence. Thus, to maintain a balance in brain network properties, local network functionality gradually declines.

Furthermore, the analysis of inter‐network FC revealed increased computerized cognitive training‐dependent FC changes in the connections between the DMN and SMN, as well as between the LN and ECN at the 6‐month visit. Although the remaining inter‐network connectivity results were not significant, they still demonstrated an increasing trend in FC with computerized cognitive training. These results indicate stronger functional coupling between these networks as a result of computerized cognitive training in patients with VCIND. These findings suggest that computerized cognitive training may enhance the coordination between networks responsible for different cognitive functions, such as language processing and executive control. This trend suggests that the brain may be adapting and becoming more interconnected in response to cognitive training, even if the changes are not yet statistically significant. Previous studies on cognitive training in Alzheimer's disease (AD) and older adults have also reported elevated FC in various brain regions within brain networks, which aligns with our study's findings.^36^, ^37^ It is noteworthy that our study divided different functional brain networks and further explored the changes in the FC attributes of the network, suggesting that the global brain network connectivity attributes increased significantly with computerized cognitive training. Furthermore, the SMN and the LN, functioning as primary networks, are responsible for controlling sensorimotor and language processing functions, respectively.^38^, ^39^ In contrast, the DMN and the ECN, considered higher cognitive networks, play pivotal roles in the resting state and cognitive task execution. The DMN is associated with self‐reflection, introspection, recall, future planning, and emotional processing, while the ECN regulates higher cognitive functions, including attention, working memory, planning, and flexibility.^40^, ^41^ Maintaining a balance between primary and higher networks is crucial for sustaining normal cognitive function.^42^ The significant increases in DMN‐SMN and ECN‐LN FC may suggest a strong association between primary and advanced network functions in response to computerized cognitive training. The causal and convergent relationships between the higher cognitive network and the primary network require further exploration through studies with larger sample sizes.

### 4.2 Alterations of the language network

Language deficits are a significant clinical feature of VCIND,^43^ yet there has been limited research on the LN in VCIND. This study identified a significant improvement in BNT scores following computerized cognitive training, indicating enhancements in language function. In this study, different from the results of FC decline in other intra‐networks, there were longitudinal increases in FC within the LN at Week 7, shedding light on the vital role of the LN in the context of computerized cognitive training interventions.

Specifically, our findings observed significant FC increases between different pairs of LN regions, including P1‐P3 and M3‐T2 at Week 7 and P2‐T3 and M2‐P3 at Month 6. M2 and M3 are located in the middle frontal gyrus and play roles in language processing, including functions related to speech processing and semantic comprehension. P1, P2, and P3 are situated in the upper and lower parietal lobules, areas that are involved in functions related to language comprehension, language expression, and speech processing. T2 and T3 are located in the brain's temporal lobe and are closely associated with language comprehension and semantic processing.^32^, ^44^ The relationships between these regions are highly intricate, as they interact within the LN to support various aspects of language function.^45^, ^46^ Different study populations and research contexts may lead to distinct connections and activities among these regions. Through in‐depth exploration of the interrelationships among these regions, we can gain a better understanding of how the language functions of patients with VCIND change with cognitive training interventions, providing valuable insights for more effective disease management. Enhanced FC within the LN suggests that computerized cognitive training can have a positive impact on language processing and related functions in patients with VCIND. In addition, the observed associations between changes in FC within the LN and neuropsychological changes further emphasize the clinical significance of these findings. Longitudinal changes in FC were significantly correlated with improvements in cognitive performance, particularly in measures such as the MoCA and the BNT. This suggests that enhanced FC within the LN is not only a neural adaptation but also has real‐world implications for the cognitive abilities of patients with VCIND. The positive correlation between FC changes and improvements in cognitive function highlights the potential therapeutic value of cognitive training programs targeting the LN in patients with VCIND.

Moreover, as mentioned in the previous section, a robust increase in FC between the LN and the ECN at Month 6 was also discovered. This increase in functional coupling reflects a stronger interaction between language and executive control functions in patients with VCIND after computerized cognitive training. In addition, there have been studies in patients with subcortical VCIND, which is the object of this study, showed a greater reduction in connections within the orbitofrontal cortex and dorsolateral prefrontal cortex.^47^ These regions, known as the main components of the prefrontal/subcortical circuit, were found to be disrupted, leading to executive dysfunction. As the disease progresses and deficits multiply, other cognitive functions are gradually impaired, including language function.^43^ Thus, this provides further support for the existing connectivity between the LN and ECN. Moreover, the causal relationship between executive function and language function to some extent explains the asynchrony in the results of intra‐ECN and intra‐LN FC after computerized cognitive training. This may be attributed to the fact that computerized cognitive training initially improves executive function deficits, which in turn alleviates language function impairments.

## 5 LIMITATIONS

Our work must be considered in light of the following limitations. First, due to the rigorous screening conditions of the experiment, our sample size is comparable to that of previous cross‐sectional FC studies and longitudinal multimodal neuroimaging studies in patients with VCIND focusing on resting‐state fMRI studies. However, it provides adequate power only for detecting moderate to large effect sizes. Larger samples may be necessary to identify more subtle relationships involving intervention measures, connectivity, and cognition. Second, while this study revealed the longitudinal alterations in FC among the networks of patients with VCIND with computerized cognitive training and suggested the FC correlation between primary motor networks and higher cognitive networks, additional methodological validation, such as effect connectivity^48^ or stepwise FC,^49^ is warranted. Third, there is substantial evidence indicating that patients with VCIND exhibit subtle yet widespread deficits in fractional anisotropy of white matter.^22^, ^50^ Therefore, the topological organization of anatomical connectivity may be another potential biomarker for VCIND.

## 6 CONCLUSION

In summary, the findings of this study highlight the potential benefits of computerized cognitive training in patients with VCIND. The cognitive training intervention resulted in improvements in certain cognitive measures, particularly in global cognitive functioning and language ability. These improvements were associated with specific changes in FC within the LN and its connections with other networks. These results provide insights into the neuroplasticity and adaptive changes that occur with cognitive training in patients with VCIND, shedding light on the underlying mechanisms and potential therapeutic implications for this population. However, further research is needed to confirm and expand upon these findings, as well as to explore the long‐term effects of cognitive training in VCIND.

## AUTHOR CONTRIBUTIONS

X. F, Y. T, and J. L made substantial contributions to the conception and design of the work. Q. L analyzed the data and wrote the full manuscript. Y. X screened the subjects and collected data Z. Z analyzed the data and modified the manuscript. All the authors have read and approved the final version of the manuscript.

## CONFLICT OF INTEREST STATEMENT

All the authors declare that there are no conflicts of interest.

## Supporting information


Data S1

## Data Availability

The datasets generated during and/or analyzed during the current study are available from the corresponding author upon reasonable request.
