# Supplementary material for: Effects of computerized cognitive training on functional brain networks in patients with vascular cognitive impairment and no dementia
Source: CNS Neurosci Ther. 2024 Jun 3;30(6):e14779. doi: 10.1111/cns.14779 (PMC11145123; doi:10.1111/cns.14779)
Supplement: Supplementary file 1 — Data S1 [file CNS-30-e14779-s001.docx]

**Supplementary Information**

**SI Methods**

**Functional brain network construction**

The sparsity threshold, S, ranged from 0.10 to 0.5, with an interval of 0.05. We adopted a sparsity threshold to alleviate the effects of thresholding on the final topological metrics^1^ and because this strategy has been shown to be less influenced by subjects’ head motion ^2^.

**Network metrics**

Global efficiency (Eq. (1)) was defined as how efficiently the whole network exchanges information computed as:

$E_{global}^{G}=\frac{1}{n}\sum_{i\in N} E_{i}^{G}=\frac{1}{n}\sum_{i\in N} \frac{\sum_{j\in N, j\neq i} {(d_{ij}^{G})}^{-1}}{n-1}$ (1)

where E_i_ was node i’s weighted efficiency and d_ij_^G^ was the shortest weighted path length between nodes i and j. d_ij_^G^ was defined as:

$d_{ij}^{G}=\sum_{a_{uv}\in g_{i\leftrightarrow j}^{G}} f(G_{uv})$ (2)

where a_uv_ was the connection status; a_ij_ = 1 as a link (i, j) existed (i.e., node i and j are neighbors); otherwise a_ij_ = 0 (a_ii_ = 0 for all i). G_uv_ stood for the connection weights between nodes u and v. f corresponded to a map from weight to length (i.e., an inverse mapping). g_i_↔︎j^G^ denoted the shortest weighted path between node i and j. N was the set of all nodes, and the number of nodes in the given network was denoted by the letter n. The superscript “G” indicated that this metric was calculated with weighted networks. Unless otherwise stated, all metrics used in the present study were weighted, so the superscript “G” was omitted in other sections.

Local efficiency was defined as the mean efficiency of the local subnetworks^3^ and computed as:

$E_{local}^{G}=\frac{1}{2}\sum_{i\in N} E_{local, i}^{G}=\frac{1}{2}\sum_{i\in N} \frac{\sum_{j,h\in N, j\neq i} {{({G_{ij}G_{ih}[d}_{jh}^{G}\left( N_{i} \right)]}^{-1})}^{1/3}}{k_{i}(k_{i}-1)}$(3)

where E_local_, i^G^ was the weighted local efficiency of node i. The connection weights between nodes i and j were denoted by G_ij_, and d_jh_^G^(N_i_) was the weighted length of the shortest path between j and h, composed exclusively of the neighbors of i.

**SI Results**

To verify the robustness of our findings, we also computed local efficiency and global efficiency. As Table S1 showed, the overall results were largely confirmed. We found training group showed significantly lower local efficiency values (P = 0.030) at week 7, while an increased trend of global efficiency (P>0.05).

Table S1 Longitudinal local efficiency and global efficiency for training group and control group

| Variables | Change from baseline to week 7 | | | Change from the baseline to month 6 | | |
| --- | --- | --- | --- | --- | --- | --- |
|  | Training group  (95% CI) | Control group  (95% CI) | *P* (group x time) | Training group  (95% CI) | Control group  (95% CI) | *P* (group x time) |
| Local efficiency | 3.356  (1.467 to 5.244) | -0.085  (-2.062 to 1.892) | **0.030** | 2.224  (0.256–4.192) | 1.358  (20.899 to 3.614) | 0.501 |
| Global efficiency | 0.003  (-0.003 to 0.007) | -0.018  (-0.058 to 0.022) | 0.676 | -0.003  (-0.010 to 0.005) | -0.032  (-0.086 to 0.022) | 0.658 |

**References**

1. Fornito A, Zalesky A, Breakspear M. Graph analysis of the human connectome: promise, progress, and pitfalls. Neuroimage 2013; 80: 426-444.

2. Yan CG, Craddock RC, He Y, Milham MP. Addressing head motion dependencies for small-world topologies in functional connectomics. Frontiers in human neuroscience 2013; 7: 910.

3. Latora V, Marchiori M. Efficient Behavior of Small-World Networks. Physical Review Letters 2001; 87(19): 198701.
